# Interleukin-12 Is Associated with Rheumatoid Factor and Interferon-γ, but Not with Disease Activity, in Patients with Rheumatoid Arthritis

**DOI:** 10.3390/diagnostics16213643

**Published:** 2026-11-04

**Authors:** Iván Ferraz-Amaro, Juan Carlos Quevedo-Abeledo, Enrique García-Barrera, Luisa María Villar, Javier Gonzalo Ocejo-Vinyals, Miguel Ángel González-Gay

**Affiliations:** 1 Department of Internal Medicine, University of La Laguna (ULL), 38071 Tenerife, Spain; 2 Division of Rheumatology, Hospital Universitario de Canarias, 38320 Tenerife, Spain; 3 Instituto de Investigación Sanitaria de Canarias (IISC), 38320 Tenerife, Spain; quevedojcarlos@yahoo.es; 4 Division of Rheumatology, Hospital Doctor Negrín, 35010 Las Palmas de Gran Canaria, Spain; 5 Department of Immunology, Hospital Ramón y Cajal, 28034 Madrid, Spain; egbarrera@salud.madrid.org (E.G.-B.); luisamaria.villar@salud.madrid.org (L.M.V.); 6 Department of Immunology, Hospital Universitario Marqués de Valdecilla, Instituto de Investigación Sanitaria Marqués de Valdecilla (IDIVA), 39008 Santander, Spain; javiergonzalo.ocejo@scsalud.es; 7 Division of Rheumatology, IIS-Fundación Jiménez Díaz, 28015 Madrid, Spain; 8 Department of Medicine and Psychiatry, University of Cantabria, 39005 Santander, Spain

**Keywords:** Interleukin 12, rheumatoid arthritis, disease activity, inflammation, cardiovascular disease

## Abstract

**Background:** IL-12 is a proinflammatory cytokine produced mainly by activated dendritic cells and macrophages that drives the differentiation of naïve T cells into Th1 cells and stimulates interferon-γ production by natural killer and T cells, thereby linking innate and adaptive immunity. Ultrasensitive quantification of IL-12 has not previously been performed in large rheumatoid arthritis (RA) cohorts, mainly due to the limited availability and high cost of these platforms. In the present study, we measured serum IL-12 using an ultrasensitive assay to systematically assess its relationship with key disease characteristics in a large, well-characterized cohort of patients with RA. **Methods:** A total of 216 patients with RA were recruited and underwent a comprehensive assessment of disease characteristics and disease activity. Lipid parameters and hematological markers were assessed, insulin resistance indices were calculated, and metabolic syndrome was defined according to established criteria. Arterial stiffness, carotid intima-media thickness, and plaque presence were evaluated through carotid ultrasonography. Serum IL-12 levels were quantified using Simoa (Single Molecule Array) technology. To examine the relationship between clinical and metabolic parameters and IL-12 concentrations, multivariable linear regression models were applied. **Results:** In multivariable analyses, serum IL-12 levels were independently associated with IFN-γ and rheumatoid factor positivity. However, no significant relationships were found between IL-12 and disease activity indices, acute-phase reactants, cardiovascular risk factors, subclinical atherosclerosis, lipid profile, glucose metabolism, or hematological parameters. **Conclusions:** Using an ultrasensitive assay, we found that serum IL-12 levels were associated with interferon-γ levels and rheumatoid factor positivity in patients with RA, supporting a role for IL-12 at the interface between cellular and humoral immunity. In contrast, circulating IL-12 did not appear to be a useful systemic biomarker of disease activity or of the cardiovascular and hematological comorbidities associated with RA.

## 1. Introduction

IL-12 is a heterodimeric pro-inflammatory cytokine consisting of two covalently linked subunits, p35 and p40, which together form the biologically active p70 heterodimer. It is mainly produced by antigen-presenting cells, especially macrophages and dendritic cells, which release it in response to pathogens, bacterial products, and intracellular parasites [1]. One of the most prominent functions of IL-12 is its ability to stimulate interferon-gamma (IFN-γ) production by natural killer cells, T cells, B cells, and even antigen-presenting cells themselves. In turn, IFN-γ activates phagocytic cells and promotes cell-mediated immunity [2]. In addition, IL-12 promotes the differentiation of naïve CD4+ T cells into T helper 1 cells, which play an essential role in defense against intracellular pathogens. This process occurs through STAT4 activation and induction of the transcription factor T-bet [1]. Other functions include enhancing the proliferation and cytotoxic activity of both natural killer cells and cytotoxic T lymphocytes, as well as inducing an antiangiogenic program mediated by IFN-γ-inducible genes [3]. Accordingly, IL-12 plays a clinically relevant role in three major domains: host defense against intracellular pathogens, antitumor immunity, and the promotion of pathogenic Th1 responses in autoimmune diseases such as Crohn’s disease and psoriatic arthritis, where blockade of the shared IL-12/IL-23 p40 subunit with agents such as ustekinumab has become an established therapeutic strategy [4].

IL-12 has been implicated in the pathogenesis of rheumatoid arthritis (RA). In this regard, in a study that measured serum and synovial fluid IL-12 (p70) levels in 152 patients with RA, 69 patients with osteoarthritis, and 50 healthy controls, detectable serum IL-12 concentrations were found in 42.2% of RA patients, compared with only 1.4% of osteoarthritis patients and 10% of healthy controls [4]. In addition, IL-12 levels correlated with tender and swollen joint counts and C-reactive protein [4]. In another study in which IL-12 levels were evaluated in serum and synovial fluid from 64 patients with RA and 25 with osteoarthritis, IL-12 levels were significantly higher in RA [5]. In this study, both serum and synovial IL-12 levels also correlated positively with the disease activity index. In addition, basic research studies have demonstrated that IL-12 transcripts and heterodimer levels were higher in synovial fluid than in peripheral blood, suggesting a predominant Th1 response [6], and that IL-12 protein is expressed by macrophages and synovial lining cells in RA, as shown by immunohistochemistry [7].

However, a major limitation of these previous studies is that IL-12 circulates at extremely low concentrations, and with conventional ELISA a substantial proportion of samples are undetectable. As noted above, only 42.2% of RA patients had detectable serum IL-12 by conventional ELISA [4]. This has led to concerns that the results of previous studies may not be fully accurate and may have been influenced by this limitation. With the advent of ultrasensitive techniques such as Simoa (Single Molecule Array), the determination of IL-12 is now possible in serum at the femtogram/mL level. This would help to more accurately characterize how IL-12 relates to disease characteristics.

Based on previous evidence implicating IL-12 in Th1-mediated synovial inflammation and its reported association with RA disease activity, we hypothesized that serum IL-12 concentrations, when quantified using an ultrasensitive assay, would be detectable in most patients with RA and would be associated with markers of disease activity and systemic inflammation. Accordingly, the primary objective of this study was to quantify serum IL-12 concentrations using the ultrasensitive Simoa platform in a large, well-characterized cohort of patients with RA and to determine their independent association with predefined measures of disease activity and acute-phase reactants. Secondary objectives were to evaluate the exploratory associations of serum IL-12 concentrations with autoantibody status, disease-related characteristics, traditional cardiovascular risk factors, and metabolic syndrome. To the best of our knowledge, this is the first study to assess serum IL-12 levels using ultrasensitive technology in patients with RA.

## 2. Material and Methods

### 2.1. Study Participants

This was a cross-sectional study that included 216 consecutively recruited RA patients, all of whom were 18 years or older and met the 2010 ACR/EULAR classification criteria [8]. Patients were recruited from two tertiary-care hospitals: Hospital Universitario de Canarias and Hospital Universitario de Gran Canaria Dr. Negrín. They had been diagnosed by rheumatologists and attended regular follow-up appointments at rheumatology outpatient clinics. For the purpose of inclusion in the present study, the duration of RA disease was required to be ≥1 year. Participants were excluded if they had: (1) current or previous malignancy; (2) an active infection at enrolment; (3) hypothyroidism; (4) established cardiovascular disease, defined as a history of myocardial infarction, angina requiring revascularization, heart failure, clinically relevant valvular heart disease, or arrhythmia requiring treatment; (5) chronic respiratory disease, including chronic obstructive pulmonary disease, persistent asthma requiring maintenance treatment, interstitial lung disease, or chronic respiratory failure; or (6) nephrotic syndrome, defined as nephrotic-range proteinuria with hypoalbuminemia. These criteria were established a priori and were assessed from medical records and the baseline clinical evaluation. Besides, given the common use of glucocorticoids in RA, patients receiving prednisone at a dose greater than 10 mg/day, or an equivalent dose of another glucocorticoid, were excluded to minimize potential confounding effects. Therefore, only patients with a dose of prednisone ≤ 10 mg/day were recruited. A detailed participant flow chart is provided in the Supplementary Figure S1. The study was approved by the Institutional Review Committees at Hospital Universitario de Canarias and Hospital Universitario Doctor Negrín (approval no. 2023-48), and all participants provided informed written consent. Research was conducted in accordance with relevant guidelines and the Declaration of Helsinki.

### 2.2. Data Collection

Participants underwent a comprehensive examination, which included a questionnaire on cardiovascular risk factors and medication use. The physical examination assessed body mass index (BMI), abdominal circumference, and blood pressure (both systolic and diastolic) under standardized conditions. Information on smoking, diabetes, and hypertension was collected, and specific diagnoses and medication details were verified through medical record reviews.

Disease activity in patients with RA was measured using the Disease Activity Score (DAS28) in 28 joints [9], the Clinical Disease Activity Index (CDAI) [10] and the Simple Disease Activity Index (SDAI) [11]. DAS28-erythrocyte sedimentation rate (ESR) and DAS28-C-reactive protein (CRP) were classified into distinct categories based on predefined thresholds: remission (<2.6), low (>2.6 to 3.2), moderate (>3.2 to 5.1), or high disease activity (>5.1) as previously described [12]. Likewise, the SDAI categories were defined as follows: remission (≤3.3), low disease activity (>3.3 and ≤11), moderate disease activity (>11 and ≤26), and high disease activity (>26). Similarly, the CDAI was categorized into remission (≤2.8), low disease activity (>2.8 and ≤10), moderate disease activity (>10 and ≤22), and high disease activity (>22). These categorizations adhere to established criteria [13].

The presence of metabolic syndrome was determined using the National Cholesterol Education Program (NCEP/ATPIII) criteria [14]. According to the NCEP ATP III definition, metabolic syndrome is diagnosed when three or more of the following five criteria are met: waist circumference exceeding 102 cm in men or 88 cm in women, blood pressure greater than 130/85 mmHg, fasting triglyceride levels above 150 mg/dL, fasting high-density lipoprotein (HDL) cholesterol levels lower than 40 mg/dL in men or 50 mg/dL in women, and fasting blood glucose levels exceeding 100 mg/dL [14]. The Systematic Coronary Risk Evaluation-2 (SCORE2) CV risk tool was calculated as previously described using age, gender, smoking status, systolic blood pressure, and non-HDL-cholesterol [15]. SCORE2 estimates an individual’s 10-year risk of fatal and non-fatal CV disease events in individuals aged 40 to 69 years. For healthy people aged ≥70 years, the SCORE2-OP (older persons) algorithm estimates 5-year and 10-year fatal and nonfatal CV disease events.

### 2.3. Laboratory Assessments

Cholesterol, triglycerides, and HDL-cholesterol were measured using the enzymatic colorimetric assay in serum. Lipoproteins were assessed using a quantitative immunoturbidimetric assay in serum. Cholesterol ranged from 0.08 to 20.7 mmol/L (intra-assay coefficient of variation of 0.3%); triglycerides ranged from 4 to 1.000 mg/dL (intra-assay coefficient of variation of 1.8%); and HDL-cholesterol ranged from 3 to 120 mg/dL (intra-assay coefficient of variation of 0.9%). The atherogenic index was calculated using the total cholesterol: HDL-cholesterol ratio according to the Castelli formula. Low-density lipoprotein (LDL)-cholesterol was calculated using the Friedewald formula. The erythrocyte sedimentation rate (ESR) was determined using the Westergren method. High-sensitivity C-reactive protein (hsCRP) levels were measured using a high-sensitivity immunoassay.

The homeostatic model assessment (HOMA) method was performed to determine IR. Briefly, the HOMA model enabled an estimate of insulin sensitivity (%S) and β-cell function (%B) from fasting plasma insulin, C peptide, and glucose concentrations. In this study we used HOMA2, the updated-computer HOMA model [16]. This model can be used to assess insulin sensitivity and β-cell function from paired fasting plasma glucose and specific insulin, or C peptide, concentrations across a range of 1–2200 pmol/L for insulin and 1–25 mmol/L for glucose. C peptide better estimates β-cell function since it is a marker of secretion; and insulin data is preferable when calculating %S since HOMA-%S is derived from glucose disposal as a function of insulin concentration. In our study, IR and %S were calculated using insulin serum levels. Otherwise, %B was calculated using C-peptide serum levels. The computer model provided a value for insulin sensitivity expressed as HOMA2-%S (in which 100% is normal). HOMA2-IR (insulin resistance index) is simply the reciprocal of %S. Insulin (Architect Abbott, 2000I, Madrid, Spain) and C peptide (Immulite 2000, Siemens, Munich, Germany) were determined by chemiluminescent immunometric assays.

Blood cell counts were measured using the Sysmex-XN automated blood cell analyzer (Sysmex, Kobe, Japan). This instrument uses the standard deviation, rather than the coefficient of variation, of the mean corpuscular volume distribution curve to calculate the red cell distribution width. IFN-α was measured from serum samples using the Simoa (Single Molecule Array) IFN-α MS Advantage PLUS KitTM (Quanterix, Billerica, MA, USA). IFN-γ were measured using the Cytokine 4-Plex C Advantage PLUS Reagent KitTM (Quanterix, Billerica, MA, USA) following the manufacturer’s instructions. Sample processing and analysis were done using an HD-X analyzer (software version 4.16.2307.14001; Quanterix). IL-12 was measured Simoa^®^ IL-12p70 Advantage PLUS Kit (Quanterix Corporation, Billerica, MA, USA), limit of detection (LOD) was 0.001–0.006 pg/mL. None of the patients had undetectable IL-12 levels.

### 2.4. Carotid Ultrasound Assessment

A carotid ultrasound was performed to assess the thickness of the carotid intima-media wall (cIMT) in the common carotid artery. The goal was to identify any localized plaques in the extracranial carotid arteries, located outside the skull (extracranial carotid tree). The measurements were carried out using the EsaoteMylab 70 ultrasound system from Genova, Italy. This system is equipped with a 7–12 MHz linear transducer and employs the Quality Intima Media Thickness in real-time (QIMT) automated software-guided radiofrequency technique developed by Esaote in Maastricht, Holland. The assessment process followed the guidelines established by the Mannheim Consensus [17], which establishes criteria for identifying plaques within the accessible extracranial carotid arteries. These arteries include the common carotid artery, the bulb, and the internal carotid artery. Plaque criteria were established as the presence of a localized bulge within the arterial lumen, with a measurement of cIMT exceeding >1.5 mm. Additionally, the bulge needed to be at least 50% larger than the adjacent cIMT or result in an arterial lumen reduction of >0.5 mm [17].

Carotid arterial stiffness was assessed using high-resolution ultrasound imaging with an Esaote^®^ system equipped with radiofrequency-based echo-tracking technology. The Esaote echo-tracking software automatically calculated key stiffness parameters, including pulse velocity wave (PVW), augmentation index (AIx), distensibility coefficient, compliance, and the β-stiffness index, based on simultaneous recordings of arterial diameters and brachial blood pressure. All measurements were synchronized with the electrocardiogram to ensure accurate timing of systolic and diastolic phases. This standardized protocol allowed for the reliable and reproducible assessment of carotid stiffness, providing valuable insights into early vascular alterations associated with CV risk [18].

### 2.5. Statistical Analysis

Demographic and clinical data for patients with RA were summarized using means and standard deviations, or percentages for categorical variables. Continuous variables that did not follow a normal distribution were reported as medians with interquartile ranges (IQR). The adequacy of the sample size was evaluated assuming a linear relationship between DAS28-ESR and log-transformed serum IL-12 levels. With a two-sided alpha of 0.05 and 80% statistical power, a cohort of around 190 patients is sufficient to detect a small-to-moderate effect, corresponding to a correlation coefficient near 0.20. On this basis, the sample available in the study was deemed adequate for the primary analysis. Missing data were addressed by carefully reviewing all collected variables for completeness prior to analysis. Cases with missing key clinical or laboratory data were excluded from specific analyses to avoid introducing bias. The extent of missing data was minimal, and details of missing data are transparently reported. Distribution of IL-12 values was assessed visually using histograms and normal Q-Q plots in both the original and log-transformed scales. Because raw IL-12 concentrations showed a markedly right-skewed distribution with substantial deviation from normality, log-transformed IL-12 values were used in subsequent parametric analyses. The distribution of the log-transformed variable showed improved symmetry and a closer approximation to normality (Supplementary Figure S2). Associations between log-transformed circulating IL-12 and disease characteristics in patients with RA were evaluated using multivariable linear regression. The assumption of normally distributed residuals in the linear regression models was assessed by visual inspection of histograms and normal Q-Q plots, together with the Shapiro–Wilk test. For back-transformation of beta coefficients, exp(beta) should be calculated; percentage change may be estimated as (exp(beta) − 1) × 100. Potential confounders were identified using a change-in-estimate approach. Specifically, variables were considered confounders and retained in the multivariable model if their inclusion resulted in a change of 10% or more in the beta coefficient of the main exposure variable. This criterion was applied iteratively, adjusting the model to control for confounding while maintaining parsimony. The initially selected potential confounders included age, sex and the presence of diabetes. All the analyses used a 5% two-sided significance level and were performed using Stata software, version 17/SE (StataCorp, College Station, TX, USA). *p*-values < 0.05 were considered statistically significant. Data visualizations were performed using Julius AI (Julius AI Inc., San Francisco, CA, USA; https://julius.ai), utilizing Python 3.x.

## 3. Results

### 3.1. Demographics and Disease-Related Data of Patients with RA

A total of 216 patients with RA were included in the study. Circulating IL-12 levels showed a median of 251 (IQR 155–391) fg/mL. The demographic and clinical characteristics of the cohort are shown in Table 1. Participants had a mean age of 59 ± 9 years, and the majority were female (83%). The mean body mass index was 31 ± 18 kg/m^2^. Traditional cardiovascular risk factors were highly prevalent. In this regard, 23% of patients were active smokers, 13% had diabetes mellitus, and 41% had hypertension. Furthermore, 56% fulfilled criteria for metabolic syndrome, while 35% and 10% were receiving statins and aspirin, respectively. The median SCORE2 cardiovascular risk was 4.2 (IQR 2.4–6.2), with 57% of patients classified as low risk, 35% as intermediate risk, and 8% as high risk. Regarding subclinical atherosclerosis, the mean carotid intima-media thickness was 721 ± 141 µm, and carotid plaques were detected in 54% of the cohort (Table 1**)**.

The mean disease duration was 13 ± 9 years. At the time of evaluation, median hs-CRP levels were 2.3 mg/L (IQR 0.2–5.0), while ESR averaged 16 ± 14 mm in the first hour. Rheumatoid factor was present in 84% of patients, and 74% tested positive for anti-citrullinated protein antibodies ACPA. Disease activity indices indicated moderate disease burden, with a DAS28-ESR of 3.2 ± 1.3 and a DAS28-CRP of 2.8 ± 1.2. The median SDAI and CDAI scores were 8 (IQR 5–16) and 7 (IQR 3–14), respectively. Regarding treatment, 36% of patients were receiving prednisone, and 88% were on at least one conventional disease-modifying antirheumatic drug (DMARD), most commonly methotrexate (69%). Anti–tumor necrosis factor therapy was used by 19% of the cohort. Detailed information on other treatments and historical disease characteristics is summarized in Table 1, while data on lipid profile, insulin resistance indices, and hematological parameters are provided in Supplementary Table S1.

### 3.2. Disease Features and Their Association with IL-12 Levels

In the univariable linear regression analyses, higher serum log IL-12 levels were associated with older age and a higher abdominal-to-hip ratio. Among cardiovascular variables, aspirin use was associated with higher log IL-12 levels, whereas smoking status, obesity, hypertension, diabetes mellitus, and statin use were not related to IL-12 concentrations (Table 2).

**Figure 1:**
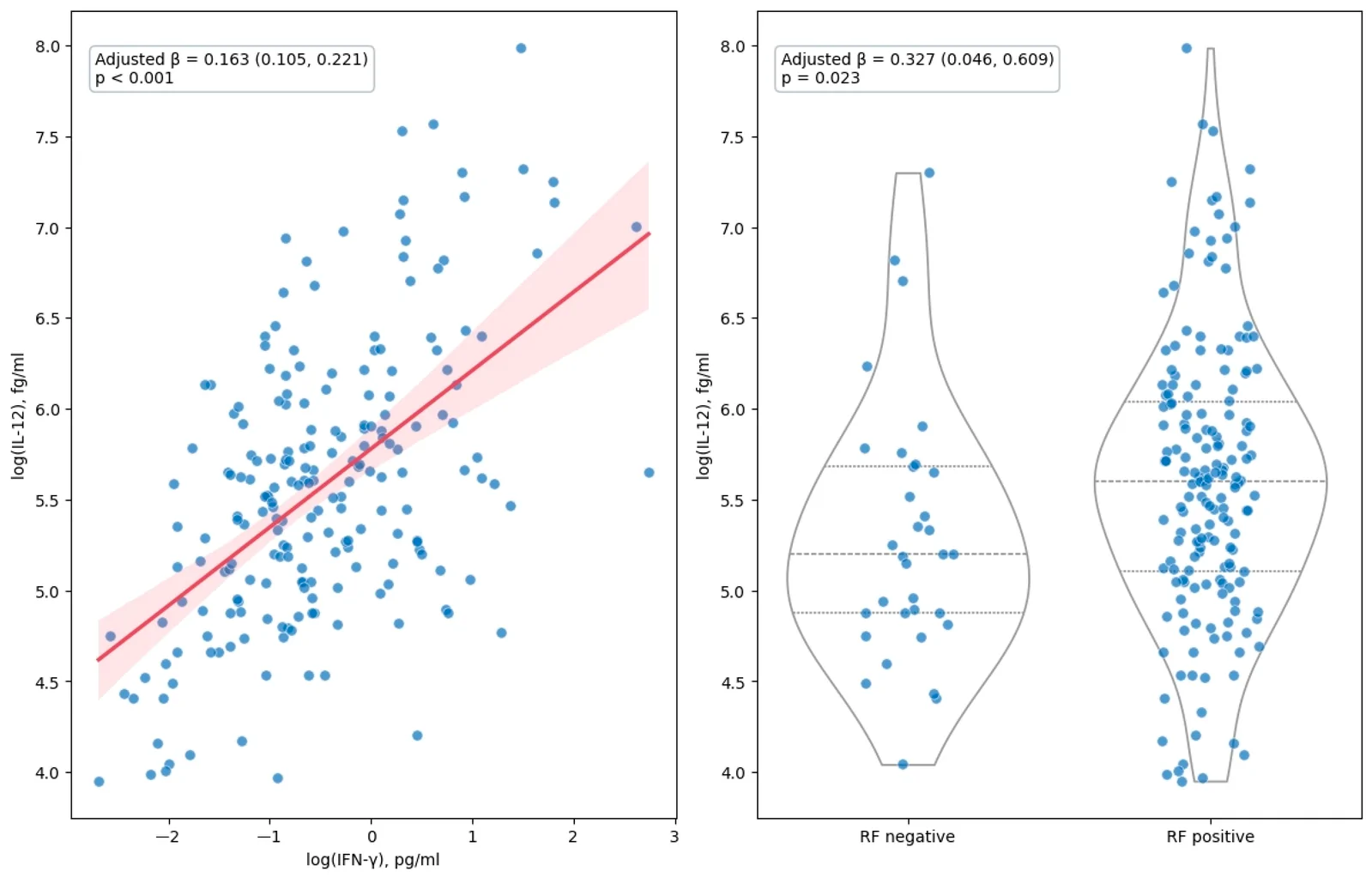
**Associations of interferon-γ and rheumatoid factor with serum IL-12 levels.** (**Left**) panel, scatter plot showing the association between log-transformed interferon-γ concentrations and log-transformed serum IL-12 levels. The red line represents the fitted linear regression line, and the shaded area indicates its 95% confidence interval. (**Right**) panel, violin plots showing the distribution of log-transformed IL-12 levels according to rheumatoid factor status. Points represent individual participants; violin plots show the distribution and internal quartiles. Beta coefficients, 95% confidence intervals, and *p* values were estimated using linear regression models adjusted for age, sex, and diabetes mellitus.

Regarding disease-related characteristics, higher interferon-γ levels and rheumatoid factor positivity were linked to higher log IL-12 levels in univariable analyses. These relationships remained significant after adjustment for age, sex, and diabetes mellitus. Specifically, interferon-γ showed the strongest relationship with IL-12 levels, with an adjusted β coefficient of 0.163 (95% CI 0.105, 0.221; *p* < 0.001). In addition, rheumatoid factor positivity was also independently related to higher IL-12 levels after adjustment (adjusted β 0.327, 95% CI 0.046, 0.609; *p* = 0.023) (Figure 1).

Disease duration, CRP, ESR, interferon-α, ACPA positivity, and continuous measures of disease activity, including DAS28-ESR, DAS28-CRP, SDAI, and CDAI, showed no significant relationship with serum IL-12 levels. Likewise, IL-12 levels did not differ significantly across disease-activity categories defined by DAS28-ESR, DAS28-CRP, SDAI, or CDAI, using remission as the reference category. Moreover, when the analyses were restricted to patients with moderate-to-high disease activity, no statistically significant associations were observed between disease activity scores and circulating IL-12 levels (Supplementary Table S2).

With regard to current treatments, prednisone use, methotrexate, anti-TNF therapy, and tocilizumab were related to IL-12 levels in univariable analyses. After multivariable adjustment, anti-TNF therapy remained independently linked to higher IL-12 levels, with an adjusted β coefficient of 0.401 (95% CI 0.144, 0.658; *p* = 0.002). No significant relationships were found for NSAIDs, conventional DMARD use, leflunomide, hydroxychloroquine, sulfasalazine, rituximab, abatacept, baricitinib, or tofacitinib (Table 2).

### 3.3. Associations of Cardiovascular Comorbidities with Serum IL-12 Levels in Patients with RA

No significant associations were observed between serum IL-12 levels and metabolic syndrome, SCORE2, or SCORE2 cardiovascular-risk categories. Similarly, carotid ultrasound parameters, including pulse wave velocity, augmentation index, and carotid plaque, were not associated with log IL-12 levels. Although cIMT showed a borderline positive association in the univariable analysis, this association was attenuated after adjustment for age, sex, and diabetes mellitus and was no longer statistically significant (Table 3).

Within the lipid profile, no significant relationships were observed between IL-12 levels and total cholesterol, triglycerides, LDL-cholesterol, non-HDL cholesterol, lipoprotein(a), apolipoprotein A1, apolipoprotein B, the Apo B:Apo A1 ratio, or the atherogenic index. HDL-cholesterol showed a borderline inverse association with IL-12 levels in both univariable and multivariable analyses. Finally, none of the glucose metabolism or insulin resistance measures, including fasting glucose, insulin, C-peptide, HOMA2-IR, HOMA2-S%, and HOMA2-B%-C-peptide, was linked to serum IL-12 levels (Table 3).

### 3.4. Relationship Between Hematological Profile and IL-12 Values

After adjustment for age, sex, and diabetes mellitus, none of the hematological parameters remained statistically significantly associated with log IL-12 levels. Hematocrit and red cell distribution width showed the closest associations to statistical significance. Higher hematocrit was related to lower IL-12 levels, with an adjusted β coefficient of −0.030 (95% CI −0.061, 0.001; *p* = 0.054). Conversely, higher red cell distribution width was associated with higher IL-12 levels, with an adjusted β coefficient of 0.067 (95% CI −0.001, 0.135; *p* = 0.055). Among leukocyte subsets, no significant relationships with IL-12 were observed. Specifically, neutrophil, lymphocyte, and basophil counts showed inverse but non-significant associations after multivariable adjustment. Platelet count was not linked to IL-12 levels, and mean platelet volume showed a borderline inverse association that did not reach statistical significance after adjustment (Table 4).

## 4. Discussion

In this study, we used an ultrasensitive Simoa-based immunoassay to characterize serum IL-12 levels in a large, well-characterized cohort of patients with RA. Our main findings indicate that serum IL-12 levels were positively associated with interferon-γ and to rheumatoid factor positivity, consistent with a role for IL-12 at the interface between cellular and humoral immunity in RA. In contrast, IL-12 was not related to disease activity, acute-phase reactants, disease duration, cardiovascular risk factors, subclinical atherosclerosis, or hematological parameters. To our knowledge, this is the first study to quantify circulating IL-12 using an ultrasensitive technique in patients with RA, thereby overcoming the detection limitations of conventional assays that have constrained previous research in this field.

The positive association between circulating IL-12 and interferon-γ in our cohort of patients with RA is biologically consistent with the central role of IL-12 in promoting Th1 immune responses. Produced primarily by antigen-presenting cells, IL-12 is a key inducer of interferon-γ secretion by T lymphocytes and natural killer cells, thereby amplifying cellular immunity. The relationship between IL-12 and IFN-γ has previously been described in patients with RA and in healthy individuals. However, none of these studies used ultrasensitive techniques to measure both cytokines. Furthermore, evidence supports a relationship between IL-12 and IFN-γ in RA, both in serum and in synovial tissue [4,19]. Although the IL-12/IFN-γ axis is active in RA, particularly within the synovium, it does not appear to be the dominant pathogenic driver proposed by the classical Th1 model of the disease. The role of IFN-γ in RA remains incompletely understood. This uncertainty is further supported by findings from a cohort of 216 patients, in which serum IFN-γ levels were not associated with most clinical manifestations or disease activity, in contrast to IFN-α [20].

Moreover, the relationship between IL-12 and rheumatoid factor positivity found in the study is also of potential interest, as it suggests that IL-12 may contribute not only to cellular immune activation but also to the regulation of humoral autoimmune responses. While rheumatoid factor production is generally considered a B cell-driven phenomenon, autoreactive B cell responses are strongly influenced by T cell assistance and cytokine networks in which IL-12 may play an indirect role. Besides, IL-12 promotes the differentiation and expansion of Th1 cells and stimulates IFN-γ production. In turn, IFN-γ may contribute to an inflammatory milieu that enhances B-cell activation, T-cell–B-cell interactions, antigen presentation, and autoantibody production. However, this interpretation remains speculative, and the cross-sectional design precludes conclusions regarding causality. In addition, RF positivity is not specific to RA and may occur in other autoimmune diseases, chronic infections, and healthy older individuals. Although active infection, hepatitis C infection, malignancy, or other autoimmune diseases were excluded, residual confounding by unmeasured conditions cannot be excluded. Taken together, these findings support the concept that IL-12 has a strategic position at the interface between cellular and humoral immunity in RA.

The lack of associations between IL-12 and measures of disease activity, acute-phase reactants, or disease duration suggests that circulating IL-12 is not a major determinant of the inflammatory burden detected by conventional clinical and laboratory indices in established RA. This observation may reflect the compartmentalized nature of IL-12 biology, where local cytokine activity within inflamed synovial tissue is not adequately reflected in circulating concentrations. Furthermore, current treatment strategies may attenuate systemic inflammation without fully normalizing the underlying immunological pathways linked to IL-12 production. Consequently, serum IL-12 may be more indicative of a specific immunological phenotype characterized by Th1 activation and autoantibody production than of overall disease severity. In this regard, a phase II study evaluating ustekinumab (an anti-IL-12/23 p40 monoclonal antibody) and guselkumab (an anti-IL-23 monoclonal antibody) in patients with active RA despite methotrexate treatment showed no significant difference in ACR20 response rates at week 28 between the combined ustekinumab group (53.6%), the combined guselkumab group (41.3%), and the placebo group (40.0%) [21]. This negative result suggests that, unlike in psoriatic arthritis, blocking the IL-12/23 pathway is not an effective therapeutic strategy in RA. Consistent with the findings of this clinical trial, our study found no association between serum IL-12 levels and disease activity, whether assessed by composite indices of disease activity, acute-phase reactants, or disease duration.

Taken together, these observations suggest that circulating IL-12 is not a useful biomarker of disease activity in RA, at least at the systemic level. However, these findings do not completely exclude a possible contribution of IL-12 to disease pathogenesis at the cellular or molecular level, which might not be adequately reflected in serum measurements.

Regarding the T positive and significant relationship between IL-12 and rheumatoid factor positivity using Simoa ultrasensitive technique in our cohort, in a former study that included 140 patients with RA, rheumatoid factor was found to be moderately correlated with IL-12 levels [22]. Moreover, in a separate study that measured plasma IL-12 levels in 90 patients with RA and 90 healthy controls, IL-12 concentrations were significantly higher in rheumatoid factor-positive patients than in rheumatoid factor-negative RA patients [23]. Moreover, a review of cytokines as biomarkers in RA disclosed that IL-12 levels were elevated before the onset of clinically manifest disease in individuals who subsequently developed RA, particularly among those with a positive rheumatoid factor [24]. Taken together, these observations support a role for IL-12 at the interface between cellular (Th1) and humoral (autoantibody-mediated) immunity in RA, highlighting its potential contribution to the development of humoral autoimmunity.

In healthy subjects, a reciprocal relationship links IL-12 and interferon-γ within a feedback loop that forms one of the key pillars of Th1-mediated cellular immunity, connecting the innate and adaptive arms of the immune system [2]. In fact, IL-12’s main function is to stimulate interferon-γ secretion from natural killer cells, CD4+ and CD8+ T cells, dendritic cells, and macrophages. Notably, the relationship between these two cytokines operates as a bidirectional amplification loop: IL-12 secreted by antigen-presenting cells stimulates interferon-γ production by natural killer and T cells, which in turn enhances the capacity of macrophages and dendritic cells to further upregulate IL-12 secretion, thereby closing a self-reinforcing positive feedback circuit [2]. This relationship has been previously demonstrated to persists in RA. In this regard, serum and synovial fluid IL-12 (p70) levels in patients with RA have been shown to correlate positively with interferon-γ levels [4]. This supports the concept that IL-12 drives a Th1 response with interferon-γ production in RA. Additionally, interferon-γ and IL-12 transcripts have been reported to be increased in synovial fluid mononuclear cells compared with peripheral blood mononuclear cells [6]. In our study, in which ultrasensitive IL-12 was assessed, this relationship was preserved in patients with RA, which would suggest that this physiological function, observed in healthy individuals, is maintained in patients with RA.

Our study is the first to analyze the role of IL-12 in RA’s cardiovascular disease. IL-12 has a well-established proatherogenic role in cardiovascular disease, with evidence spanning from early atherosclerosis to acute coronary syndromes, and with demonstrated prognostic value following myocardial infarction [25]. Moreover, patients with cardiovascular disease present elevated blood IL-12 levels [26]. Despite this, we found no consistent association between IL-12 and the lipid profile, cardiovascular risk scores, metabolic syndrome or subclinical atherosclerosis in our cohort of patients with RA. This would suggest that the accelerated atherosclerosis that typically accompanies RA does not appear to be mediated by, or reflected in, serum IL-12 levels. This finding is not unexpected, given that IL-12 was not related to disease activity in our cohort, which is considered one of the main drivers of accelerated cardiovascular disease in RA [27,28].

Endogenous IL-12 plays a dual regulatory role in hematopoiesis and modulates multiple hematological parameters [29]. On one hand, when acting directly on isolated hematopoietic progenitor cells, IL-12 promotes multilineage proliferation and differentiation. On the other hand, in the presence of NK cells and T lymphocytes, IL-12 drives the production of interferon-γ and TNF-α, which synergistically inhibit hematopoietic colony formation and disrupt the bone marrow microenvironment. Consequently, dysregulation of IL-12, whether through excess or deficiency, results in clinically significant hematological abnormalities across a broad spectrum of diseases. Despite this, we found no relationship between IL-12 and the values of the three blood cell lineages.

The lack of association between IL-12 and cardiovascular risk factors, subclinical atherosclerosis, or hematological parameters further suggests that IL-12 is not a major driver of the systemic comorbidities commonly associated with RA, at least at the circulating level. While experimental studies have implicated IL-12 in atherogenesis and chronic inflammatory processes, our findings indicate that serum IL-12 concentrations do not reflect the vascular or hematological alterations observed in patients with RA.

This study is subject to several limitations that deserve to be discussed. First, we did not include a healthy control group, since our main goal was to explore the relationship between IL-12 and clinical characteristics specifically within patients with RA, rather than to compare cytokine levels with those of the general population, an aim that has already been addressed, at least in part, by previous studies. Additionally, the cross-sectional nature of our design reflects a single time point, which prevents us from establishing temporal sequence and, therefore, from drawing any causal conclusions regarding the associations identified. Third, despite the lack of association between circulating IL-12 and conventional measures of disease activity, a biologically meaningful contribution at the tissue, cellular, or transcriptomic level cannot be excluded. Fourth, the cross-sectional design of our study limits the ability to examine associations with flares or highly active disease states; given that IL-12 levels may fluctuate in parallel with changes in disease activity or therapeutic regimens, a single measurement is inherently insufficient to capture intra-individual variability over time. Besides, it is important to emphasize that the present study specifically quantifies IL-12 p70, a biologically active heterodimer formed by the covalent linkage of the p35 and p40 subunits; accordingly, our results should not be generalized to the independent serum levels of either constituent subunit. Also, another limitation of this study is the lack of data on the duration of prednisone treatment. Consequently, the potential influence of cumulative glucocorticoid exposure on the study outcomes could not be assessed. Finally, we also acknowledge that esidual confounding by concomitant therapies cannot be entirely excluded.

Despite the limitations discussed above, our study also has several notable strengths. The relatively large sample size enabled a robust multivariable analysis. In addition, our cohort was thoroughly characterized, with detailed information available not only on disease-related variables but also on hematological parameters and cardiovascular risk factors. Lastly, the application of the Simoa platform provided a highly sensitive method of quantification, and IL-12 was detectable in all participants included in the study.

In conclusion, using an ultrasensitive Simoa-based assay for the first time in RA, we show that serum IL-12 correlates with interferon-γ and rheumatoid factor positivity in patients with RA, but is not related to disease activity, cardiovascular risk, or hematological parameters. These findings suggest that, at the systemic level, circulating IL-12 does not behave as a useful biomarker of disease activity or associated comorbidities in RA, although a relevant role at the cellular or tissue level cannot be excluded.

## Figures and Tables

**Table 1:** Demographics, cardiovascular risk factors, and disease related data in patients with RA.

	Rheumatoid Arthritis	Missing Data
	(n = 216)	n (%)
IL-12, femtog/mL	251 (155–391)	6 (3)
Age, years	59 ± 9	0 (0)
Female, n (%)	179 (83)	0 (0)
BMI, kg/m^2^	29 ± 5	0 (0)
Abdominal circumference, cm	96 ± 14	1 (0)
Hip circumference, cm	104 ± 11	1 (0)
Abdominal to hip ratio	0.92 ± 0.10	1 (0)
Cardiovascular data		
CV risk factors, n (%)		
Current smoker	49 (23)	0 (0)
Obesity	86 (40)	0 (0)
Hypertension	88 (41)	0 (0)
Diabetes Mellitus	29 (13)	0 (0)
Metabolic syndrome, n (%)	119 (56)	4 (2)
Statins, n (%)	79 (35)	1 (0)
Aspirin, n (%)	21 (10)	0 (0)
SCORE2, %	4.4 (2.4–6.2)	7 (3)
Low to moderate	124 (57)	
High	75 (35)	
Very high	17 (8)	
Carotid ultrasound		
Pulse wave velocity, m/s	7.6 ± 1.7	42 (19
Augmentation index (Aix), %	7.7 (3.6–11.8)	74 (34)
cIMT, mm	0.721 ± 0.141	6 (3)
Carotid plaque, n (%)	116 (54)	0 (0)
Disease related data		
Disease duration, years	13 ± 9	0 (0)
CRP at time of study, mg/L	2.3 (0.2–5.0)	7 (3)
ESR at time of study, mm/1º hour	18 ± 14	2 (1)
IL-6, pg/mL	5.8 (3.6–11.5)	7 (3)
Interferon alpha, femtog/mL	51 (19–122)	12 (6)
Interferon gamma, femtog/mL	538 (349–1140)	7 (3)
Rheumatoid factor, n (%)	180 (84)	2 (1)
ACPA, n (%)	157 (74)	4 (2)
DAS28-ESR	3.2 ± 1.3	2 (1)
Remission	83 (39)	
Low disease activity	36 (17)	
Moderate disease activity	73 (34)	
High disease activity	22 (10)	
DAS28-CRP	2.8 ± 1.2	0 (0)
Remission	106 (49)	
Low disease activity	31 (14)	
Moderate disease activity	73 (34)	
High disease activity	6 (3)	
SDAI	8 (5–16)	0 (0)
Remission	31 (14)	
Low disease activity	91 (42)	
Moderate disease activity	69 (32)	
High disease activity	25 (12)	
CDAI	7 (3–14)	0 (0)
Remission	53 (25)	
Low disease activity	85 (39)	
Moderate disease activity	54 (25)	
High disease activity	24 (11)	
History of extraarticular manifestations, n (%)	52 (24)	3 (1)
Erosions, n (%)	91 (44)	9 (4)
Current drugs, n (%)		
Prednisone	78 (36)	0 (0)
Prednisone doses, mg/day	5 (2.5–5)	0 (0)
NSAIDs	80 (37)	0 (0)
DMARDs	191 (88)	0 (0)
Methotrexate	150 (69)	0 (0)
Leflunomide	43 (20)	0 (0)
Hydroxychloroquine	9 (4)	0 (0)
Salazopyrin	4 (2)	0 (0)
Anti TNF therapy	42 (19)	0 (0)
Tocilizumab	8 (4)	0 (0)
Rituximab	10 (5)	0 (0)
Abatacept	11 (5)	0 (0)
Baricitinib	4 (2)	0 (0)
Tofacitinib	5 (2)	0 (0)

Data represent means ± SD or median (IQR) when data were not normally distributed. Prednisone denotes using prednisone equivalent to a dose of ≤10 mg/day. CV: cardiovascular; LDL: low-density lipoprotein; HDL: high-density lipoprotein; CRP: C reactive protein; NSAID: Nonsteroidal anti-inflammatory drugs; DMARD: disease-modifying antirheumatic drug; TNF: tumor necrosis factor; Obesity; ESR: erythrocyte sedimentation rate; BMI: body mass index; DAS28: Disease Activity Score in 28 joints; ACPA: Anti-citrullinated protein antibodies; HOMA: homeostatic model assessment; CDAI: Clinical Disease Activity Index; SDAI: Simple Disease Activity Index; cIMT: carotid intima media thickness.

**Table 2:** Relationship of disease characteristics to IL-12 serum levels.

	*log* IL-12 fg/mL			
	Beta Coef. (95%CI), *p*			
	Univariable	*p*	Multivariable	*p*
Age, years	**0.015 (0.003, 0.027)**	**0.012**		
Female	−0.048 (−0.327, 0.231)	0.73		
BMI, kg/m^2^	−0.011 (−0.032, 0.010)	0.31		
Abdominal circumference, cm	0.002 (−0.006, 0.010)	0.58		
Hip circumference, cm	−0.006 (−0.016, 0.004)	0.21		
Abdominal to hip ratio	**1.196 (0.110, 2.283)**	**0.031**		
Cardiovascular data				
CV risk factors				
Current smoker	0.001 (−0.249, 0.251)	0.99		
Obesity	−0.054 (−0.269, 0.162)	0.62		
Hypertension	−0.019 (−0.233, 0.196)	0.87		
Diabetes Mellitus	−0.225 (−0.538, 0.087)	0.16		
Statins	0.069 (−0.151, 0.289)	0.54		
Aspirin	**0.432 (0.061, 0.803)**	**0.023**		
Disease related data				
Disease duration, years	0.010 (−0.003, 0.022)	0.12	0.008 (−0.005, 0.020)	0.23
CRP at time of study, mg/L	0.001 (−0.006, 0.007)	0.83		
ESR at time of study, mm/ 1st hour	0.004 (−0.003, 0.011)	0.30		
Interferon alpha, pg/mL	0.039 (−0.022, 0.101)	0.21		
Interferon gamma, pg/mL	**0.163 (0.104, 0.221)**	**<0.001**	**0.163 (0.105, 0.221)**	**<0.001**
Rheumatoid factor	**0.306 (0.020, 0.593)**	**0.036**	**0.327 (0.046, 0.609)**	**0.023**
ACPA	0.159 (−0.080, 0.398)	0.19	0.178 (−0.058, 0.413)	0.14
DAS28-ESR	0.029 (−0.050, 0.107)	0.47		
Remission	ref.			
Low disease activity	0.159 (−0.147, 0.465)	0.31		
Moderate and high disease activity	0.141 (−0.092, 0.374)	0.23		
DAS28-CRP	0.006 (−0.085, 0.097)	0.90		
Remission	ref.			
Low disease activity	0.158 (−0.155, 0.471)	0.32		
Moderate and high disease activity	0.048 (−0.182, 0.278)	0.68		
SDAI	0.001 (−0.006, 0.007)	0.85		
Remission	ref.			
Low disease activity	−0.066 (−0.389, 0.257)	0.69		
Moderate and high disease activity	0.021 (−0.299, 0.341)	0.90		
CDAI	0.002 (−0.010, 0.014)	0.70		
Remission	ref.			
Low disease activity	0.014 (−0.257, 0.286)	0.92		
Moderate and high disease activity	0.043 (−0.231, 0.317)	0.76		
History of extraarticular manifestations	0.100 (−0.146, 0.346)	0.42		
Erosions	0.193 (−0.023, 0.408)	0.079		
Current drugs				
Prednisone	**−0.233 (−0.449, −0.017)**	**0.034**	−0.201 (−0.415, 0.013)	0.065
Prednisone doses, mg/day	−0.058 (−0.122, 0.006)	0.075	−0.053 (−0.122, 0.017)	0.13
NSAIDs	0.105 (−0.112, 0.321)	0.34		
DMARDs	−0.013 (−0.350, 0.324)	0.94		
Methotrexate	**0.239 (0.012, 0.467)**	**0.039**	0.201 (−0.026, 0.429)	0.082
Leflunomide	−0.212 (−0.476, 0.052)	0.11	−0.179 (−0.441, 0.083)	0.18
Hydroxychloroquine	−0.298 (−0.816, 0.219)	0.26		
Salazopyrin	0.282 (−0.486, 1.050)	0.47		
Anti TNF therapy	**0.379 (0.119, 0.640)**	**0.004**	**0.401 (0.144, 0.658)**	**0.002**
Tocilizumab	**0.625 (0.045, 1.204)**	**0.035**	0.566 (−0.007, 1.139)	0.053
Rituximab	−0.346 (−0.838, 0.145)	0.17	−0.293 (−0.778, 0.192)	0.24
Abatacept	0.131 (−0.340, 0.603)	0.58		
Baricitinib	−0.225 (−0.994, 0.543)	0.56		
Tofacitinib	−0.346 (−1.034, 0.342)	0.32		

In this analysis IL-12 is the dependent variable. Multivariable-adjusted analysis included age, sex and diabetes as confounders, selected using the 10% change-in-estimate criterion for the beta coefficient. Only variables with a univariable association with *p* < 0.20 were included in the multivariable adjustment. For back-transformation, exp(beta) should be calculated; percentage change may be estimated as (exp(beta) − 1) × 100. NSAID: Nonsteroidal anti-inflammatory drugs; DMARD: disease-modifying antirheumatic drug. TNF: tumor necrosis factor; ESR: erythrocyte sedimentation rate; BMI: body mass index; DAS28: Disease Activity Score in 28 joints. ACPA: Anti-citrullinated protein antibodies. CDAI: Clinical Disease Activity Index; SDAI: Simple Disease Activity Index. Significant *p* values are depicted in bold.

**Table 3:** Relationship between cardiovascular disease features and serum IL-12 Levels.

	log IL-12 fg/mL			
	Beta Coef. (95%CI), *p*			
	Univariable	*p*	Multivariable	*p*
Metabolic syndrome	0.008 (−0.205, 0.222)	0.94		
SCORE2, %	0.020 (−0.013, 0.054)	0.23		
Low to moderate	ref.			
High	0.015 (−0.209, 0.238)	0.90		
Very high	0.011 (−0.451, 0.473)	0.96		
Carotid ultrasound				
Pulse wave velocity, m/s	0.006 (−0.065, 0.078)	0.86		
Augmentation index (Aix), %	0.002 (−0.012, 0.016)	0.77		
cIMT, mm	0.697 (−0.066, 1.460)	0.073	0.538 (−0.305, 1.380)	0.21
Carotid plaque	0.086 (−0.125, 0.296)	0.42		
Lipid profile				
Total cholesterol, mg/dL	−0.001 (−0.004, 0.002)	0.47		
Triglycerides, mg/dL	−0.000 (−0.002, 0.002)	0.86		
HDL-cholesterol, mg/dL	−0.005 (−0.012, 0.002)	0.13	−0.006 (−0.014, 0.001)	0.091
LDL-cholesterol, mg/dL	−0.000 (−0.003, 0.003)	0.94		
LDL:HDL cholesterol ratio	0.081 (−0.037, 0.200)	0.18	0.080 (−0.045, 0.205)	0.21
Non-HDL cholesterol, mg/dL	−0.000 (−0.003, 0.003)	0.89		
Lipoprotein (a), mg/dL	−0.000 (−0.001, 0.001)	0.86		
Apolipoprotein A1, mg/dL	−0.002 (−0.006, 0.001)	0.21		
Apolipoprotein B, mg/dL	0.000 (−0.004, 0.005)	0.91		
Apo B:Apo A1 ratio	0.194 (−0.204, 0.592)	0.34		
Atherogenic index	0.063 (−0.025, 0.151)	0.16	0.066 (−0.028, 0.160)	0.17
Insulin resistance indices				
Glucose, mg/dL	−0.002 (−0.006, 0.001)	0.22		
Insulin, µU/mL	0.001 (−0.003, 0.005)	0.74		
C-peptide, ng/mL	−0.003 (−0.044, 0.038)	0.90		
HOMA2-IR	−0.017 (−0.065, 0.030)	0.47		
HOMA2-S%	−0.001 (−0.003, 0.001)	0.27		
HOMA2-B%-C-peptide	0.001 (−0.000, 0.002)	0.20		

In this analysis IL-12 serum values are the dependent variable. Regression analyses for metabolic syndrome and SCORE2 were not adjusted because these variables are derived from composite calculations. cIMT: carotid intima media thickness; HOMA2: homeostatic model assessment. LDL: low-density lipoprotein; HDL: high-density lipoprotein. SCORE2: Systematic COronary Risk Evaluation 2. Multivariable-adjusted analysis included age, sex, and diabetes as confounders, selected using the 10% change-in-estimate criterion for the beta coefficient. Only variables with a univariable association with *p* < 0.20 were included in the multivariable adjustment. For back-transformation, exp(beta) should be calculated; percentage change may be estimated as (exp(beta) − 1) × 100. Significant *p* values are depicted in bold.

**Table 4:** Association between hematological parameters and serum IL-12 levels.

	log IL-12 fg/mL			
	Beta Coef. (95%CI), *p*			
	Univariable	*p*	Multivariable	*p*
Hematological profile				
Red blood cells	−0.193 (−0.438, 0.053)	0.12	−0.204 (−0.461, 0.053)	0.12
Hemoglobin, g/dL	−0.052 (−0.132, 0.028)	0.20		
Hematocrit, %	−0.023 (−0.052, 0.006)	0.12	−0.030 (−0.061, 0.001)	0.054
Mean corpuscular volume, fL	0.005 (−0.015, 0.025)	0.62		
Mean corpuscular hemoglobin, pg	0.014 (−0.036, 0.064)	0.59		
Mean corpuscular hemoglobin concentration, g/dL	0.019 (−0.079, 0.117)	0.71		
Red cell distribution width, %	**0.073 (0.006, 0.141)**	**0.033**	0.067 (−0.001, 0.135)	0.055
Leucocytes × 10^3^/mm^3^	−0.028 (−0.074, 0.018)	0.23		
Neutrophils × 10^3^/mm^3^	−0.053 (−0.112, 0.006)	0.079	−0.048 (−0.108, 0.013)	0.12
Lymphocytes × 10^3^/mm^3^	−0.091 (−0.213, 0.031)	0.14	−0.086 (−0.207, 0.034)	0.16
Monocytes × 10^3^/mm^3^	−0.151 (−0.629, 0.326)	0.53		
Eosinophils × 10^3^/mm^3^	−0.285 (−0.911, 0.341)	0.37		
Basophils × 10^3^/mm^3^	−2.380 (−5.542, 0.781)	0.14	−2.491 (−5.606, 0.623)	0.12
Platelets × 10^3^/mm^3^	0.001 (−0.001, 0.002)	0.34		
Mean platelet volume, fL	−0.059 (−0.131, 0.014)	0.11	−0.060 (−0.131, 0.012)	0.10

In this analysis IL-12 is the dependent variable. Multivariable-adjusted analysis included age, sex and diabetes as confounders, selected using the 10% change-in-estimate criterion for the beta coefficient. Only variables with a univariable association with *p* < 0.20 were included in the multivariable adjustment. For back-transformation, exp(beta) should be calculated; percentage change may be estimated as (exp(beta) − 1) × 100. Significant *p* values are depicted in bold.

## Data Availability

All data underlying the study’s findings is transparently provided in the Zenodo link: https://zenodo.org/records/22200202.

## Supplementary Materials

The following supporting information can be downloaded at: https://www.mdpi.com/article/10.3390/diagnostics16213643/s1, Table S1: Lipid profile, insulin resistance indices and hematological pattern in rheumatoid arthritis patients; Table S2: Disease activity scores relation to log IL-12 values in the moderate-to-high disease activity categories; Figure S1: Participants flow chart; Figure S2: Distributional assessment of serum IL-12 levels.

## References

[1] Zundler S., Neurath M.F. (2015). Interleukin-12: Functional Activities and Implications for Disease. Cytokine Growth Factor Rev..

[2] Trinchieri G. (2003). Interleukin-12 and the Regulation of Innate Resistance and Adaptive Immunity. Nat. Rev. Immunol..

[3] Del Vecchio M., Bajetta E., Canova S., Lotze M.T., Wesa A., Parmiani G., Anichini A. (2007). Interleukin-12: Biological Properties and Clinical Application. Clin. Cancer Res..

[4] Kim W.U., Min S.Y., Cho M.L., Youn J., Min J.K., Lee S.H., Park S.H., Cho C.S., Kim H.Y. (2000). The Role of IL-12 in Inflammatory Activity of Patients with Rheumatoid Arthritis (RA). Clin. Exp. Immunol..

[5] Petrovic-Rackov L., Pejnovic N. (2006). Clinical Significance of IL-18, IL-15, IL-12 and TNF-Alpha Measurement in Rheumatoid Arthritis. Clin. Rheumatol..

[6] Bucht A., Larsson P., Weisbrot L., Thorne C., Pisa P., Smedegård G., Keystone E.C., Grönberg A. (1996). Expression of Interferon-Gamma (IFN-Gamma), IL-10, IL-12 and Transforming Growth Factor-Beta (TGF-Beta) MRNA in Synovial Fluid Cells from Patients in the Early and Late Phases of Rheumatoid Arthritis (RA). Clin. Exp. Immunol..

[7] Sakkas L.I., Johanson N.A., Scanzello C.R., Platsoucas C.D. (1998). Interleukin-12 Is Expressed by Infiltrating Macrophages and Synovial Lining Cells in Rheumatoid Arthritis and Osteoarthritis. Cell. Immunol..

[8] Aletaha D., Neogi T., Silman A.J., Funovits J., Felson D.T., Bingham C.O., Birnbaum N.S., Burmester G.R., Bykerk V.P., Cohen M.D. (2010). 2010 Rheumatoid Arthritis Classification Criteria: An American College of Rheumatology/European League Against Rheumatism Collaborative Initiative. Ann. Rheum. Dis..

[9] Prevoo M.L.L., Van’T Hof M.A., Kuper H.H., Van Leeuwen M.A., Van De Putte L.B.A., Van Riel P.L.C.M. (1995). Modified Disease Activity Scores That Include Twenty-eight-joint Counts Development and Validation in a Prospective Longitudinal Study of Patients with Rheumatoid Arthritis. Arthritis Rheum..

[10] Smolen J.S., Breedveld F.C., Schiff M.H., Kalden J.R., Emery P., Eberl G., van Riel P.L., Tugwell P. (2003). A Simplified Disease Activity Index for Rheumatoid Arthritis for Use in Clinical Practice. Rheumatology.

[11] Aletaha D., Smolen J. (2005). The Simplified Disease Activity Index (SDAI) and the Clinical Disease Activity Index (CDAI): A Review of Their Usefulness and Validity in Rheumatoid Arthritis. Clin. Exp. Rheumatol..

[12] Aletaha D., Ward M.M., Machold K.P., Nell V.P.K., Stamm T., Smolen J.S. (2005). Remission and Active Disease in Rheumatoid Arthritis: Defining Criteria for Disease Activity States. Arthritis Rheum..

[13] Aletaha D., Martinez-Avila J., Kvien T.K., Smolen J.S. (2012). Definition of Treatment Response in Rheumatoid Arthritis Based on the Simplified and the Clinical Disease Activity Index. Ann. Rheum. Dis..

[14] (2001). Expert Panel on Detection, Evaluation, and Treatment of High Blood Cholesterol in Adults. Executive Summary of the Third Report of the National Cholesterol Education Program (NCEP) Expert Panel on Detection, Evaluation, and Treatment of High Blood Cholesterol in Adults (Adult Treatment Panel III). JAMA.

[15] (2021). SCORE2 Risk Prediction Algorithms: New Models to Estimate 10-Year Risk of Cardiovascular Disease in Europe. Eur. Heart J..

[16] Wallace T.M., Levy J.C., Matthews D.R. (2004). Use and Abuse of HOMA Modeling. Diabetes Care.

[17] Touboul P.J., Hennerici M.G., Meairs S., Adams H., Amarenco P., Bornstein N., Csiba L., Desvarieux M., Ebrahim S., Hernandez Hernandez R. (2012). Mannheim Carotid Intima-Media Thickness and Plaque Consensus (2004-2006-2011). An Update on Behalf of the Advisory Board of the 3rd, 4th and 5th Watching the Risk Symposia, at the 13th, 15th and 20th European Stroke Conferences, Mannheim, Germany, 2004, Brussels, Belgium, 2006, and Hamburg, Germany, 2011. Cerebrovasc. Dis..

[18] Laurent S., Cockcroft J., Van Bortel L., Boutouyrie P., Giannattasio C., Hayoz D., Pannier B., Vlachopoulos C., Wilkinson I., Struijker-Boudier H. (2006). Expert Consensus Document on Arterial Stiffness: Methodological Issues and Clinical Applications. Eur. Heart J..

[19] Kitagawa M., Suzuki H., Adachi Y., Nakamura H., Yoshino S., Sumida T. (2001). Interferon-Gamma Enhances Interleukin 12 Production in Rheumatoid Synovial Cells via CD40-CD154 Dependent and Independent Pathways. J. Rheumatol..

[20] Gómez-Bernal F., Quevedo Abeledo J.C., Almeida-Santiago C., Fernández-Cladera Y., García-Barrera E., Villar L.M., Largo R., Ocejo-Vinyals J.G., González-Gay M., Ferraz-Amaro I. (2026). Clinical and Serological Associations of Interferon-α and Interferon-γ in Rheumatoid Arthritis Patients. Rheumatology.

[21] Smolen J.S., Agarwal S.K., Ilivanova E., Xu X.L., Miao Y., Zhuang Y., Nnane I., Radziszewski W., Greenspan A., Beutler A. (2017). A Randomised Phase II Study Evaluating the Efficacy and Safety of Subcutaneously Administered Ustekinumab and Guselkumab in Patients with Active Rheumatoid Arthritis despite Treatment with Methotrexate. Ann. Rheum. Dis..

[22] Meyer P.W.A., Hodkinson B., Ally M., Musenge E., Wadee A.A., Fickl H., Tikly M., Anderson R. (2010). Circulating Cytokine Profiles and Their Relationships with Autoantibodies, Acute Phase Reactants, and Disease Activity in Patients with Rheumatoid Arthritis. Mediat. Inflamm..

[23] Shen L., Zhang H., Zhou X., Liu R. (2015). Association between Polymorphisms of Interleukin 12 and Rheumatoid Arthritis Associated Biomarkers in a Chinese Population. Cytokine.

[24] Burska A., Boissinot M., Ponchel F. (2014). Cytokines as Biomarkers in Rheumatoid Arthritis. Mediat. Inflamm..

[25] van der Heijden T., Bot I., Kuiper J. (2019). The IL-12 Cytokine Family in Cardiovascular Diseases. Cytokine.

[26] Ye J., Wang Y., Wang Z., Liu L., Yang Z., Wang M., Xu Y., Ye D., Zhang J., Lin Y. (2020). Roles and Mechanisms of Interleukin-12 Family Members in Cardiovascular Diseases: Opportunities and Challenges. Front. Pharmacol..

[27] Llorca J., Ferraz-Amaro I., Castañeda S., Plaza Z., Sánchez-Alonso F., García-Gómez C., González-Juanatey C., González-Gay M.Á. (2025). Disease Activity Predicts the Development of Cardiovascular Events in Patients with Rheumatoid Arthritis from the CARMA Cohort. Semin. Arthritis Rheum..

[28] Ferraz-Amaro I., Corrales A., Atienza-Mateo B., Vegas-Revenga N., Prieto-Peña D., Blanco R., González-Gay M. (2021). Moderate and High Disease Activity Predicts the Development of Carotid Plaque in Rheumatoid Arthritis Patients without Classic Cardiovascular Risk Factors: Six Years Follow-Up Study. J. Clin. Med..

[29] Bellone G., Trinchieri G. (1994). Dual Stimulatory and Inhibitory Effect of NK Cell Stimulatory Factor/IL-12 on Human Hematopoiesis. J. Immunol..

