# Supplementary material for: Interleukin-12 Is Associated with Rheumatoid Factor and Interferon-γ, but Not with Disease Activity, in Patients with Rheumatoid Arthritis
Source: Diagnostics (Basel). 2026 Nov 4;16(21):3643. doi: 10.3390/diagnostics16213643 (PMC13762042; doi:10.3390/diagnostics16213643)
Supplement: Supplementary file 1 [file diagnostics-16-03643-s001.zip › diagnostics-4502494-supplementary.pdf]

Supplementary Table S1. Lipid profile, insulin resistance indices and hematological pattern in rheumatoid arthritis patients.

	RA patients (n=216)	Missing n (%)
Analytical		
Total cholesterol, mg/dl	192 ± 39	7 (3)
Triglycerides, mg/dl	144 ± 64	7 (3)
HDL-cholesterol, mg/dl	55 ± 15	7 (3)
LDL-cholesterol, mg/dl	108 ± 34	7 (3)
LDL:HDL cholesterol ratio	2.1 ± 0.9	7 (3)
Non-HDL cholesterol, mg/dl	137 ± 36	7 (3)
Lipoprotein (a), mg/dl	27 (10-92)	7 (3)
Apolipoprotein A1, mg/dl	164 ± 31	7 (3)
Apolipoprotein B, mg/dl	94 ± 24	7 (3)
Apo B:Apo A1 ratio	0.60 ± 0.27	7 (3)
Atherogenic index	3.7 ± 1.2	7 (3)
Insulin resistance indices		
Glucose, mg/dl	95 ± 28	7 (3)
Insulin, µU/ml	11.3 (6.9-21.5)	6 (3)
C-peptide, ng/ml	3.93 ± 2.56	6 (3)
HOMA2-IR	1.5 (0.9-2.7)	10 (5)
HOMA2-S%	84 ± 61	10 (5)
HOMA2-B%-C-peptide	183 ± 76	7 (3)
Hematological profile		
Red blood cells		
Hemoglobin, g/dl	13.6 ± 1	0 (0)
Hematocrit, %	42 ± 4	0 (0)
Mean corpuscular volume, fl	93 ± 5	0 (0)
Mean corpuscular hemoglobin, pg	30 ± 2	0 (0)
Mean corpuscular hemoglobin concentration, g/dl	32 ± 1	0 (0)
Red cell distribution width, %	14 ± 2	0 (0)
Leucocytes / mm^3	7218 ± 2294	0 (0)
Neutrophils / mm^3	4048 ± 1791	0 (0)
Lymphocytes / mm^3	2188 ± 854	0 (0)
Monocytes / mm^3	608 ± 225	0 (0)
Eosinophils / mm^3	214 ± 167	0 (0)
Basophils / mm3	50 ± 33	0 (0)
Platelets x10e3 / mm^3	268 ± 74	0 (0)
Mean platelet volume, fl	10 ± 1	0 (0)

Data represent means ± SD or median (IQR) when data were not normally distributed. HDL: High-Density Lipoprotein; LDL: Low-Density Lipoprotein; HOMA2: Homeostasis Model Assessment 2; RA: Rheumatoid arthritis.

Supplementary Table S2. Disease activity scores relation to log IL-12 values in the moderate-to-high disease activity categories

	log IL-12 fg/ml		
Disease activity score	Beta coefficient	95% confidence interval	p-value
DAS28-ESR	−0.0616	−0.4125 to 0.2892	0.727
DAS28-CRP	−0.0809	−0.4029 to 0.2411	0.618
SDAI	−0.0164	−0.0649 to 0.0320	0.500
CDAI	−0.0252	−0.0910 to 0.0406	0.446

DAS28: Disease Activity Score in 28 joints; CDAI: Clinical Disease Activity Index; SDAI: Simple Disease Activity Index; IL: interleukin.

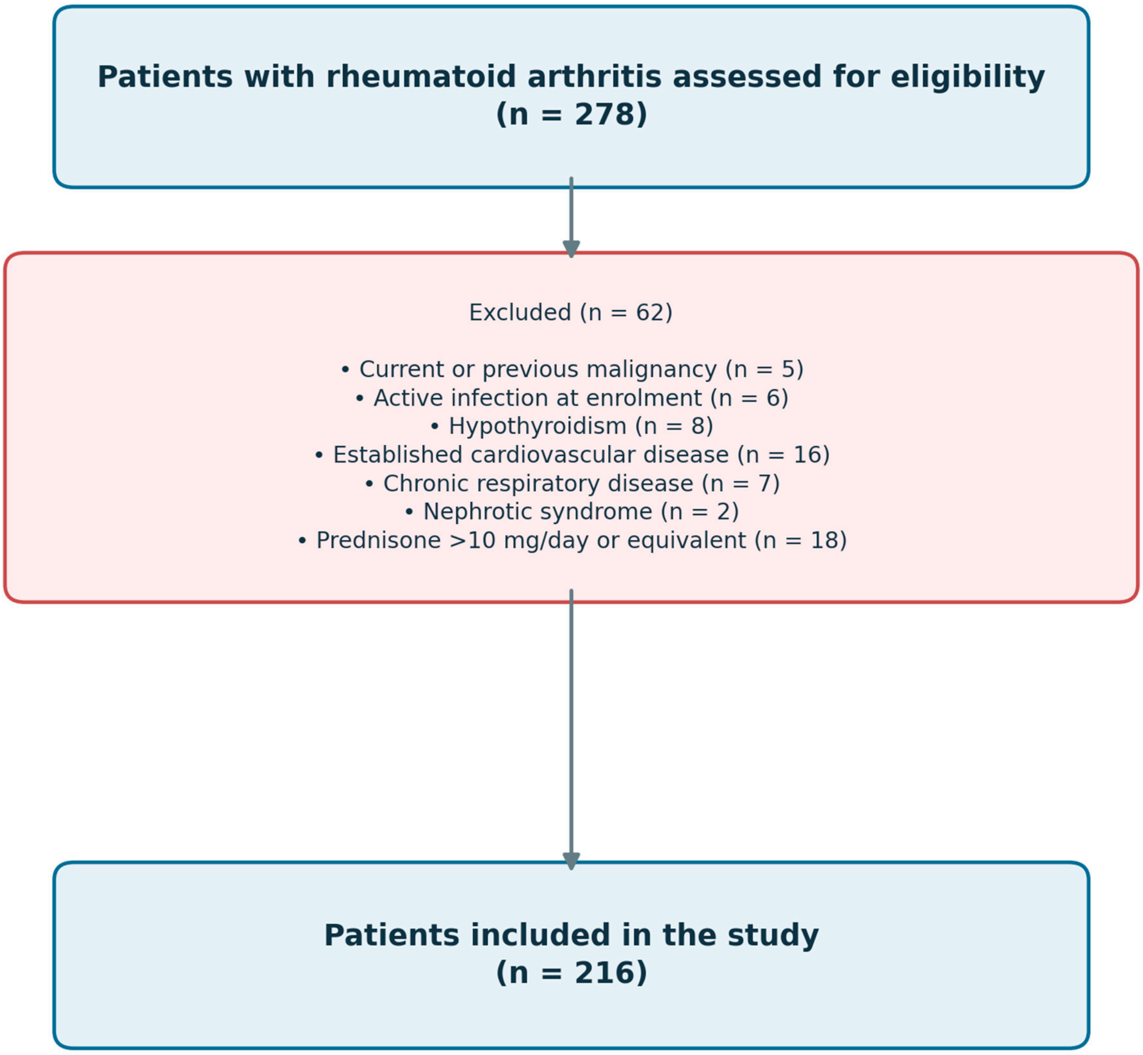

Supplementary Figure S1. Participants flow chart. A total of 278 patients with rheumatoid arthritis were assessed for eligibility. Exclusion criteria were applied sequentially: current or previous malignancy, active infection at enrolment, hypothyroidism, established cardiovascular disease, chronic respiratory disease, nephrotic syndrome, and treatment with prednisone at a dose >10 mg/day or an equivalent glucocorticoid dose. After sequential exclusions, 216 patients were included in the final analytic sample.

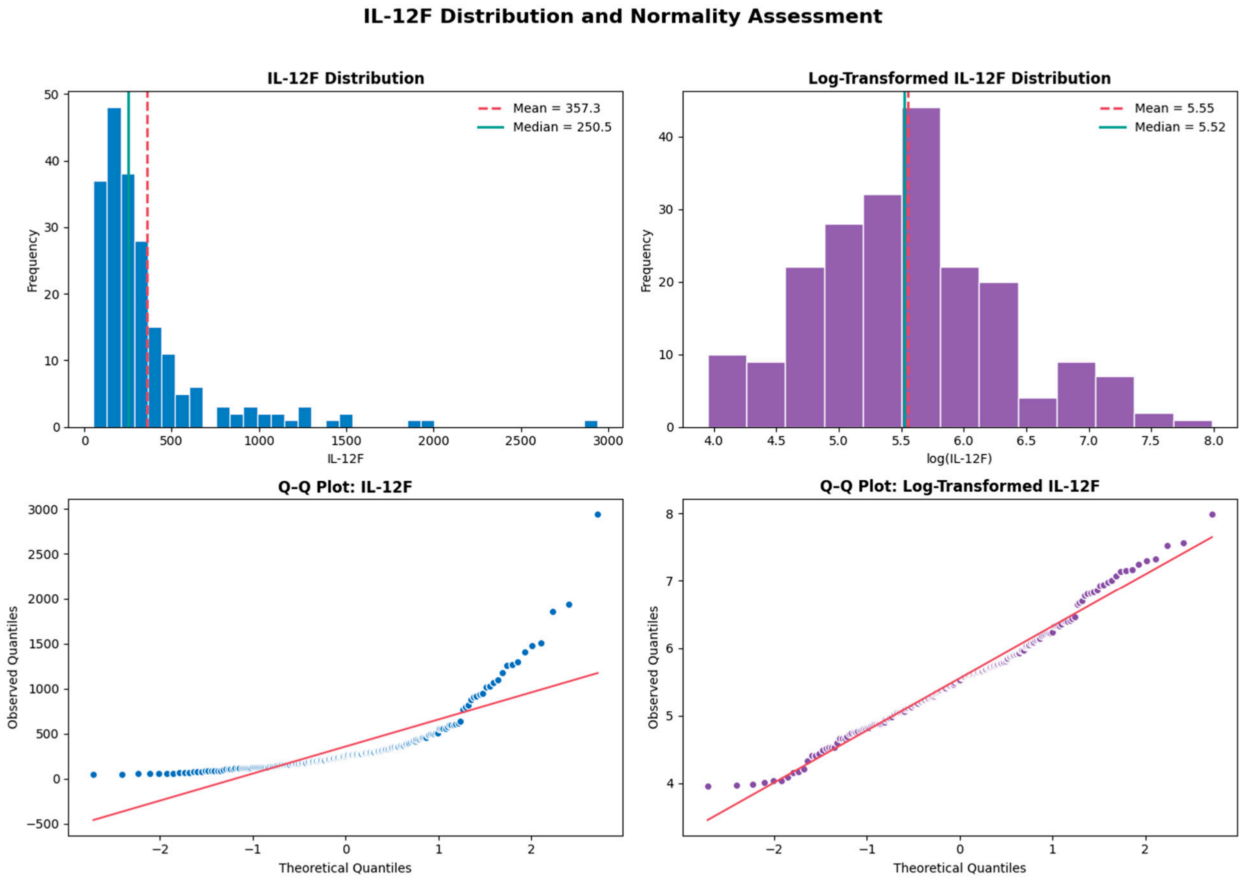

Supplementary Figure S2. Distributional assessment of serum IL-12 levels. Histograms and normal Q-Q plots for serum IL-12 in the original scale and after log transformation are presented. The untransformed variable showed substantial positive skewness, whereas the log-transformed variable demonstrated improved distributional symmetry and a better visual fit to the normal distribution.
